# 78511 Synthesis of Novel Core/Shell Polymeric Nanoparticles for Controlled Drug Release

**DOI:** 10.1017/cts.2021.657

**Published:** 2021-03-30

**Authors:** Braden Hahn

**Affiliations:** Auburn University Samuel Ginn College of Engineering

## Abstract

ABSTRACT IMPACT: This work will develop a novel drug delivery system that has improved biocompatibility and controlled release than current systems and allow for customizable loading and drug delivery to unique patient and treatment requirements. OBJECTIVES/GOALS: The goal of my project is a novel hybrid core/shell nanoparticle system for controlling in vivo chemotherapeutic concentration. The current goal is to confirm core and shell polymeric nanoparticle formation via emulsion technique and validate predictive model developed to optimize shell formation efficiency and control shell thickness. METHODS/STUDY POPULATION: Though early results are promising, they are not proof that the desired core/shell structure is being formed via my novel process. I constructed a theoretical model to use to optimize and control the process for precise shell thicknesses. Therefore, the current experimental plan focus is to not only visually confirm the predicted formation of my core/shell design but use these experiments to validate the model.

1. Gel-Suspended SEM: nanoparticles suspended in gel matrix, bisected to reveal inner structure
2. Fluorescent Conjugation Microscopy: visually-distinct dyes used to show polymer distribution and validated against the theoretical model predictions.
3. Modified Hydrophobic Dye Release: different mixtures of polymers with release showing if previous promising results due to core/shell structure RESULTS/ANTICIPATED RESULTS: As stated, the experiments will confirm the core/shell nanoparticle structure, validate the developed theoretical model, or provide direct evidence against any formation. This core/shell structure is key to the current design for controlling payload release rate and thus in vivo drug concentration. For the gel-suspension experiment, the interior core will be labeled with ultrasmall SPIONs and thus any layers within the particles will be distinct. While this result is qualitative, high magnification fluorescent microscope images will be analyzed using image processing software to determine core/shell formation efficiency and compared to estimated efficiencies from the model. Finally, the mixed release will clarify previous experiments’ release mechanism and either support or disprove shell influence. DISCUSSION/SIGNIFICANCE OF FINDINGS: The significance of this work is twofold: core/shell particles have been proven to provide variable control of release on the micron scale but not yet at the nanoscale, allowing for a circulating, targeted system that can finely control release. The process is also novel for producing this type of structure, at highly consistent quality and size.

